# Decitabine suppresses tumor growth by activating mouse mammary tumor virus and interferon-β pathways

**DOI:** 10.17305/bb.2025.12852

**Published:** 2025-08-27

**Authors:** Ryan Johnson, Andrew Brola, Cade Wycoff, William Wycoff, Seth Neumeyer, Richard Tuttle, Sarah Light, Jiayi Li, Stephen Christensen, Yingguang Liu

**Affiliations:** 1 Department of Biomedical Sciences, College of Osteopathic Medicine, Liberty University, Lynchburg, VA, USA

**Keywords:** Decitabine, DNA methyltransferase inhibitor, mouse mammary tumor virus, interferon, tumor, cancer, 4T1, MC38, interferon regulatory factor 7

## Abstract

Decitabine (DAC), a DNA methyltransferase inhibitor (DNMTi), is clinically effective in hematological malignancies such as myelodysplastic syndrome and acute myeloid leukemia, but its precise antineoplastic mechanisms remain incompletely understood. Beyond promoter demethylation, DAC is known to activate endogenous retroviruses (ERVs) and trigger type I interferon (IFN-I) responses, a phenomenon known as viral mimicry. The aim of this study was to investigate the roles of the mouse mammary tumor virus (MMTV) and interferon-β (IFN-β) in DAC-mediated tumor suppression. We employed two murine tumor models—4T1 mammary carcinoma and MC38 colon adenocarcinoma—in syngeneic immunocompetent mice, immunodeficient nude mice, and *in vitro* cultures. RNA and protein expression were assessed by quantitative PCR and immunoblotting, while functional contributions of MMTV and IFN-β were tested using short hairpin RNA (shRNA) knockdowns. DAC treatment suppressed tumor growth and pulmonary metastasis *in vivo* and inhibited cancer cell proliferation *in vitro*. It induced transcription of MMTV and expression of IFN-β, with a strong negative correlation between MMTV Env protein levels and tumor mass. Knockdown of either MMTV or IFN-β conferred resistance to DAC, confirming their functional roles. Reciprocal regulation was observed: MMTV knockdown reduced IFN-β expression, while IFN-β knockdown increased MMTV Env accumulation. Furthermore, DAC upregulated interferon regulatory factor 7 (IRF7), but this effect declined during prolonged treatment, suggesting a temporally restricted therapeutic window. In conclusion, our findings provide *in vivo* support for the viral mimicry hypothesis and demonstrate that MMTV and IFN-β contribute to DAC-mediated tumor suppression. The observed IRF7 downregulation and potential induction of immune checkpoints highlight the importance of therapeutic strategies combining DNMTis with immune checkpoint blockade to sustain antineoplastic efficacy.

## Introduction

DNA methyltransferase inhibitors (DNMTis), such as azacitidine (AZA) and decitabine (DAC), are effective treatments for specific hematological malignancies, including myelodysplastic syndrome (MDS) and acute myeloid leukemia (AML). Despite their clinical efficacy, the mechanisms underlying their action are not fully understood. Although these drugs are known to alter the expression of numerous genes by demethylating their promoters [1, 2], studies have not identified any drug-specific DNA methylation patterns or canonical target genes modified by DAC in AML cells [3]. Evidence suggests that hypomethylation may reactivate tumor-suppressor genes and induce apoptosis; however, the precise mechanisms by which DNMTis exert their effects in patients remain poorly defined [4, 5].

Beyond the direct effects of DNA hypomethylation on cell proliferation and apoptosis, DNMTis are known to modulate the immune environment in cancer patients and tumor-bearing mice [6]. Specifically, DAC has been demonstrated to suppress the expansion and function of myeloid-derived suppressor cells (MDSCs), activate CD4+ T cells, and enhance the expression of major histocompatibility complex (MHC) molecules and tumor-specific antigens, thereby synergizing with cytotoxic T lymphocytes [7–10]. Furthermore, DNMTis stimulate the expression of endogenous retroviruses (ERVs), which induce antineoplastic type I interferons (IFN-I), supporting the viral mimicry hypothesis [11]. Key sensors of double-stranded RNA (dsRNA) involved in the interferon response include Toll-like receptor 3, mitochondrial antiviral-signaling protein (MAVS), and Staufen1. Additionally, interferon regulatory factor 3 and the long noncoding RNA *TINCR* contribute to this process [11, 12]. The viral mimicry effect of DNMTis has rekindled interest in interferon-oriented cancer therapies and the mechanisms of interferon regulation within the tumor microenvironment [13]. However, much of the evidence supporting the viral mimicry hypothesis has been derived from *in vitro* studies, with a notable lack of *in vivo* mechanistic research.

The objective of this study was to investigate the phenomenon of DAC-induced viral mimicry in murine tumor models. We utilized two murine tumor cell lines: 4T1, a mouse mammary tumor cell line derived from the BALB/c strain, and MC38, a colon adenocarcinoma cell line originating from C57BL/6 mice. Both cell lines efficiently form solid tumors in their respective syngeneic host strains. The genomes of these cell lines contain endogenous proviruses of the *Mouse mammary tumor virus* (MMTV, referred to as *Mtv* for endogenous variants), which can be transmitted both endogenously through Mendelian inheritance and exogenously via maternal milk [14]. MMTV is a significant contributor to the development of mouse mammary tumors [15]. Our investigation focused on the roles of MMTV and interferon-β (IFN-β) in the antineoplastic effects of DAC. We found that DAC treatment activates MMTV expression, which subsequently induces IFN-β expression in tumor cells, ultimately resulting in reduced cell proliferation and delayed tumor growth. Notably, we observed a rapid initial upregulation of interferon regulatory factor 7 (IRF7), which gradually declined over the course of DAC treatment.

## Materials and methods

### Cell lines, culturing, and *in vitro* drug treatment

The 4T1 murine mammary cancer cell line (American Type Culture Collection; STR authenticated) was cultured in RPMI 1640 supplemented with 10% fetal bovine serum (FBS). The MC38 cell line, provided by Dr. Anthony Bauer and also STR authenticated, was maintained in Dulbecco’s Modified Eagle Medium (DMEM) with 10% FBS. All cell cultures were incubated at 37 °C in a 5% CO_2_ atmosphere.

DAC (MedChemExpress) was prepared by dissolving it in dimethyl sulfoxide (DMSO) and subsequently added to the culture media at a concentration of 100 nM. Recombinant mouse interferon-α2 (VWR) was dissolved in water and incorporated into the culture media at concentrations of 2 ng/mL and 20 ng/mL. After a treatment period of 3–4 days, cells were trypsinized and counted using a NucleoCounter NC-3000 (Chemometec), following the manufacturer’s protocols. Additionally, the cell lines were treated with BM-Cyclin to eliminate any potential mycoplasma contamination.

### Extraction of RNA, reverse transcription, and quantitative PCR

RNA extraction, reverse transcription, and real-time PCR were performed as previously described [16], using the same primers for MMTV and the reference gene *Pgk1*. Candidate housekeeping genes from the Mouse Housekeeping Gene Primer Set (RealTimePrimers) were evaluated with NormFinder [17], which identified *Pgk1* as the most stable. Primers for the *Ifnb1* gene (RealTimePrimers.com), which encodes IFN-β, were used at 200 nM. The annealing temperature for *Ifnb1* was 61 ^∘^C. All samples were quantified in triplicate wells. All quantities were calculated using the ΔΔCt method. All primer pairs had amplification efficiencies above 90%, producing a single band upon agarose gel electrophoresis and a single peak in melting point analyses.

### Plasmids, lentiviral packaging and transduction

MMTV short hairpin RNA (shRNA) plasmids were custom-designed and constructed by OriGene. Two of these plasmids targeted the *env* gene: KD1 targeted nucleotides 6394–6422, and KD3 targeted nucleotides 6720–6748, as referenced in GenBank AF033807.1. Additionally, KD2 targeted the *pol* gene, specifically nucleotides 5186–5214. Mouse *Ifnb1* shRNA plasmids were also obtained from OriGene. In both cases, 29-mer shRNA constructs were expressed using the pGFP-C-ShLenti lentiviral vector. For the control, the same vector was used to express a scrambled 29-mer shRNA. The procedures for lentiviral packaging and transduction followed the methods described in [16]. To select transduced 4T1 cells, the concentration of puromycin was gradually increased from 0.5 µg/mL to 10 µg/mL.

### Immunoblotting

Immunoblotting was conducted as outlined in [16]. Tissue samples or cultured cells were lysed using radioimmunoprecipitation assay (RIPA) buffer. The Invitrogen iBlot 2 Gel Transfer Device facilitated the transfer of proteins onto polyvinylidene difluoride (PVDF) membranes. The following primary antibodies were employed: polyclonal rabbit anti-MMTV Env (Bosterbio A30410) at a dilution of 1:2000, polyclonal rabbit anti-IFNB (MyBioSource MBS9607127) at 1:3000, polyclonal rabbit anti-IRF7 at 1:1000, and monoclonal mouse anti-glyceraldehyde-3-phosphate dehydrogenase (GAPDH) (Bosterbio M00227-6) at 1:3000. All primary antibodies were incubated overnight at 4 ^∘^C. Secondary antibodies included horseradish peroxidase (HRP)-conjugated goat anti-rabbit IgG (Bosterbio) and IRDye800CW-labeled donkey anti-mouse (LI-COR), both used at a dilution of 1:10,000. Reactions with secondary antibodies were performed at room temperature for one hour. Enhanced chemiluminescence (Bosterbio) and infrared fluorescence were captured using the ChemiDoc MP (Bio-Rad) or scanned with the Odyssey CLx Infrared Imaging System (LI-COR). Band intensities were quantified using Image Studio (LI-COR) and Image Lab (Bio-Rad) software. The quantities of target proteins were normalized to GAPDH levels to produce densitograms.

### Mouse models

Female BALB/c, C57BL/6, and NU/J mice (7–8 weeks old, with body weights of 19.2 ± 1.6 g, 18.7 ± 1.0 g, and 23.8 ± 1.4 g, respectively) were inoculated subcutaneously with 30,000 4T1 cells or 200,000 MC38 cells suspended in 50–100 µL of Versene solution (ThermoFisher), using a 30½-gauge needle at nipple number 4. Following the development of palpable tumors, typically within one week post-inoculation, the mice were stratified into treatment groups based on tumor size and body weight using the minimization method [18]. DAC was administered via subcutaneous injection every other day at doses of 0.42 mg/kg for BALB/c and NU/J mice (with the exception of the MMTV knockdown experiment, where a dose of 0.78 mg/kg was used) and 0.53 mg/kg for C57BL/6 mice. The drug was initially dissolved in DMSO, stored at –80 ^∘^C, and subsequently diluted 100-fold in phosphate-buffered saline (PBS) for injection on the back. Control mice received an equivalent volume of PBS containing 1% DMSO. Mice were weighed biweekly, and tumor volume was calculated using the formula 0.5 × length × width^2^. Experiments were concluded before tumors reached 20% of body weight or when mice exhibited signs of morbidity. Tumor mass was measured post-euthanasia. The metastatic colony count was conducted as previously described [16]. The students responsible for measuring tumors and counting colonies were blinded to group allocations during the assessments. The use of mice in this study was approved by the Institutional Animal Care and Use Committee of Liberty University (protocol numbers 70, 75, 81, 89, 96, 98).

### Statistical analysis

The two-tailed exact Mann–Whitney *U* test was employed to compare two sets of quantitative data. In instances involving multiple groups, pairwise comparisons were preplanned. Statistical significance was determined at *P* < 0.05. Correlation coefficients were calculated and assessed using the Spearman’s Rho Calculator [19]. A two-tailed post hoc power analysis was conducted with G*Power, utilizing Cohen’s d to evaluate effect sizes [20].

## Results

### DAC inhibited tumor growth and metastasis in both immunocompetent mice and nude mice

We inoculated both 4T1 and MC38 cells into the subcutaneous tissue of syngeneic mice (BALB/c and C57BL/6, respectively) and nude mice, specifically under the nipple number 4. Once the tumors became palpable, DAC was administered via subcutaneous injection every other day for a duration of 3–4 weeks. The pulmonary metastasis of 4T1 tumors was assessed by counting colonies in cell cultures following the sacrifice of the mice.

**Figure 1.**
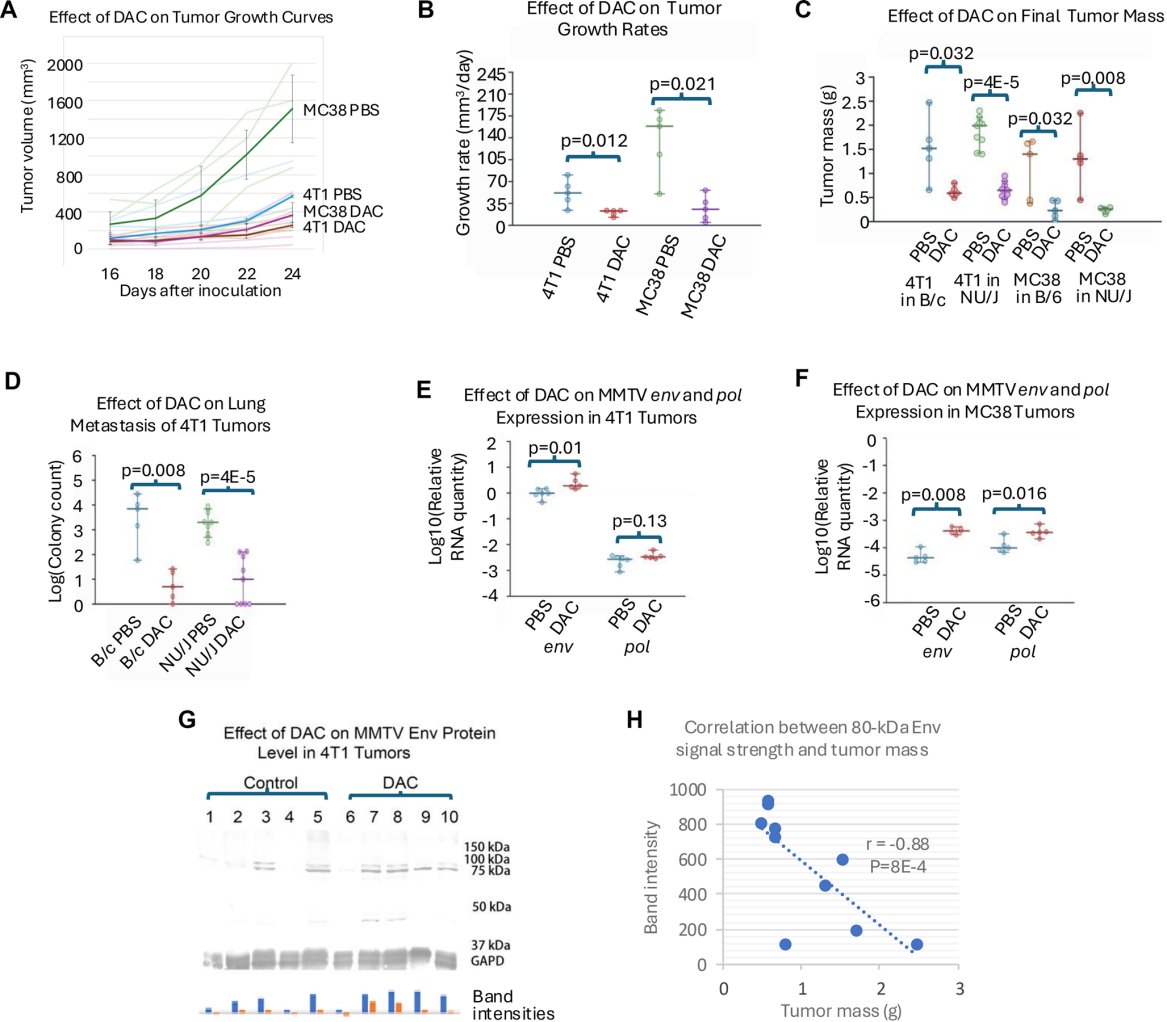
**DAC treatment, tumor growth/metastasis assessments, and MMTV expression readouts in murine tumor models.** (A) Tumor growth curves for 4T1 (BALB/c) and MC38 (C57BL/6) tumors treated with decitabine (DAC) or vehicle (PBS); *n* ═ 5 mice per group. Individual trajectories are shown with group medians overlaid (*x*-axis: Days after inoculation; *y*-axis: Tumor volume, mm^3^); (B) Tumor growth rates derived as linear-regression slopes of the curves in (A). Points indicate group medians with 95% CIs; *P* values are annotated; (C) Final tumor mass across strains and treatments: 4T1 in BALB/c and NU/J; MC38 in C57BL/6 and NU/J. *n* ═ 5 per group except 4T1 in NU/J (*n* ═ 9). Medians with 95% CIs; (D) Lung metastasis of 4T1 tumors quantified as log10 colony counts from lung cell cultures. *n* ═ 5 for BALB/c; *n* ═ 9 for NU/J. Medians with 95% CIs; (E) MMTV *env* and *pol* RNA in 4T1 tumors (BALB/c) measured by qRT-PCR and displayed as log10 relative quantity (PBS *n* ═ 6; DAC *n* ═ 5); (F) MMTV *env* and *pol* RNA in MC38 tumors (C57BL/6) measured by qRT-PCR (*n* ═ 5 per group); (G) Immunoblot of MMTV Env in 4T1 tumors: Lanes 1–5 PBS; lanes 6–10 DAC. Bands near ∼80 kDa and ∼45 kDa are shown; GAPDH (37 kDa) is the loading control. Bar plot summarizes band intensities per lane; (H) Scatter plot of tumor mass versus Env band intensity for the 10 4T1 tumors in (G); five PBS and five DAC samples. Spearman correlation statistics are annotated in the panel. Abbreviations: DAC: Decitabine (5-aza-2ʹ-deoxycytidine); PBS: Phosphate-buffered saline; MMTV: Mousemammary tumor virus; env/pol: MMTV genes; qRT-PCR: Quantitative reverse-transcription PCR; kDa: Kilodalton; CI: Confidence interval; BALB/c = B/c; C57BL/6 = B/6; NU/J: Nude mice; n: Number of mice.

DAC significantly inhibited the overall growth of both 4T1 and MC38 tumors in syngeneic and nude mice, as illustrated in Figure 1A–1C. However, the rate of primary tumor growth accelerated after 2–3 weeks of DAC treatment. Additionally, DAC effectively reduced pulmonary metastasis of the 4T1 tumor in both BALB/c and nude mice, as shown in Figure 1D.

### DAC enhanced expression of MMTV which is correlated with tumor suppression

DAC treatment of mice significantly increased the RNA expression of the *env* and *pol* genes of MMTV in both 4T1 and MC38 tumors, as determined by quantitative reverse transcription PCR (qRT-PCR) (Figure 1E and 1F). The expression levels of the MMTV Env protein varied between treated and untreated mice. A 70–80 kDa Env precursor was observed in both groups, exhibiting greater intensity in DAC-treated tumors, while a prominent 40–45 kDa band was identified exclusively in the treated group (Figure 1G). Notably, a significant negative correlation was found between Env quantity and tumor mass, with a Spearman correlation coefficient of –0.88 (Figure 1H).

### MMTV knockdown conferred resistance to DAC

**Figure 2.**
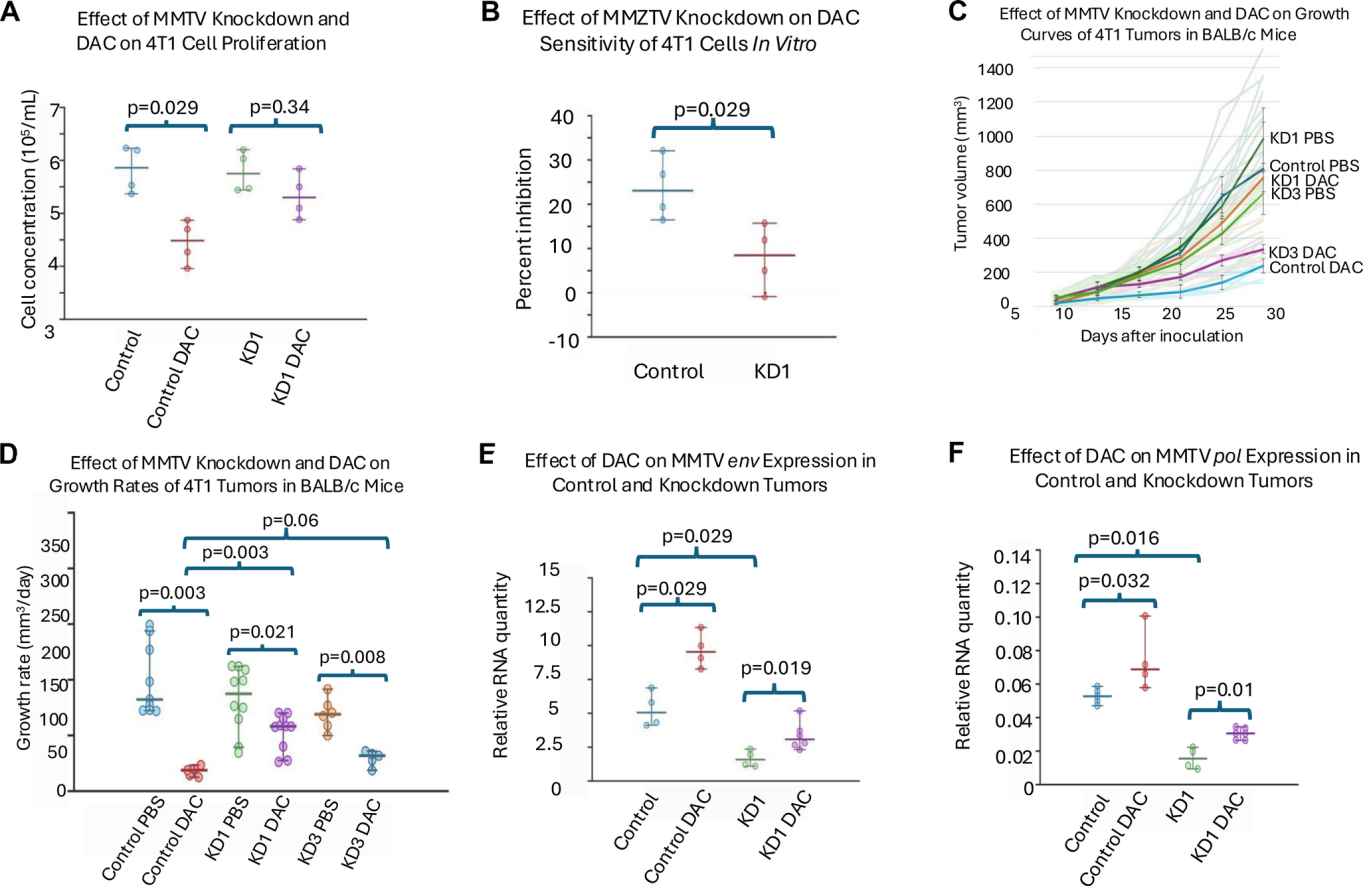
**MMTV knockdown reduces sensitivity of 4T1 cells and tumors to DAC treatment.** (A) Effect of MMTV knockdown and DAC treatment on proliferation of 4T1 cells. Cell viability was determined by NucleoCounter analysis after DAC exposure; (B) Effect of MMTV knockdown on DAC-mediated growth inhibition of 4T1 cells *in vitro*. Percent inhibition was calculated relative to untreated controls; (C) Impact of MMTV knockdown and DAC on tumor growth kinetics of 4T1 tumors in BALB/c mice. Tumor volumes were monitored over time following inoculation and treatment with PBS or DAC; (D) Growth rate analysis of 4T1 tumors in BALB/c mice after MMTV knockdown and DAC treatment. Pairwise comparisons were performed between PBS- and DAC-treated groups, as well as between control and knockdown tumors; (E) Quantification of MMTV *env* transcript levels in control and knockdown tumors with or without DAC treatment. Relative expression was determined by qRT-PCR; (F) Quantification of MMTV *pol* transcript levels in control and knockdown tumors with or without DAC treatment. Relative expression was determined by qRT-PCR. Values represent group medians with 95% confidence intervals. *n* ═ 4 for *in vitr*o assays (A and B); *n* ═ 9 for control tumors, *n* ═ 10 for KD1, and *n* ═ 5 for KD3 *in vivo* experiments (C and D); *n* ═ 4–6 for transcript quantification (E and F). Abbreviations: MMTV: Mouse mammary tumor virus; DAC: Decitabine; PBS: Phosphate-buffered saline; KD: Knockdown; qRT-PCR: Quantitative reverse transcription polymerase chain reaction.

To investigate the role of MMTV in DAC-mediated tumor suppression, we constructed lentiviral plasmids expressing shRNA that targeted the MMTV *env* and *pol* genes, which were then transduced into 4T1 cells. The knockdown of MMTV resulted in increased resistance of 4T1 cells to DAC *in vitro*, as evidenced by a higher number of surviving cells in the presence of DAC compared to the control, as indicated by viable cell counts using the NucleoCounter (Figure 2A and 2B). This trend was also observed *in vivo* (Figure 2C and 2D). Among the knockdown cell lines, KD1 exhibited a more efficient MMTV knockdown than KD3 (see Table 1 for knockdown efficiencies; KD2 was excluded due to low efficiency). Correspondingly, tumors from the KD1 cell line demonstrated greater resistance to DAC (Figure 2C and 2D).

**Table 1:** Knockdown efficiencies (percent reduction in mRNA and protein)

**Cell line**	**mRNA**	**Protein**
MMTV KD1	81.6	83
MMTV KD3	76	69.8
*Ifnb1* KD1	79.4	69.1
*Ifnb1* KD2	84.7	80.1

Abbreviations: MMTV: Mouse mammary tumor virus; *Ifnb1:* Interferon beta 1; KD: Knockdown (KD1, KD2, KD3 denote distinct knockdown lines); mRNA: Messenger RNA.

To further elucidate the role of MMTV in DAC treatment, we quantified *env* and *pol* transcripts in knockdown cell lines both in the presence and absence of DAC (Figure 2E and 2F). The RNA expression levels of *env* and *pol* were significantly diminished in the knockdown cell lines compared to the control groups. Notably, the DAC-induced upregulation of MMTV transcription was limited in the knockdown cells.

### DAC upregulated expression of IFN-β in tumor cells

**Figure 3.**
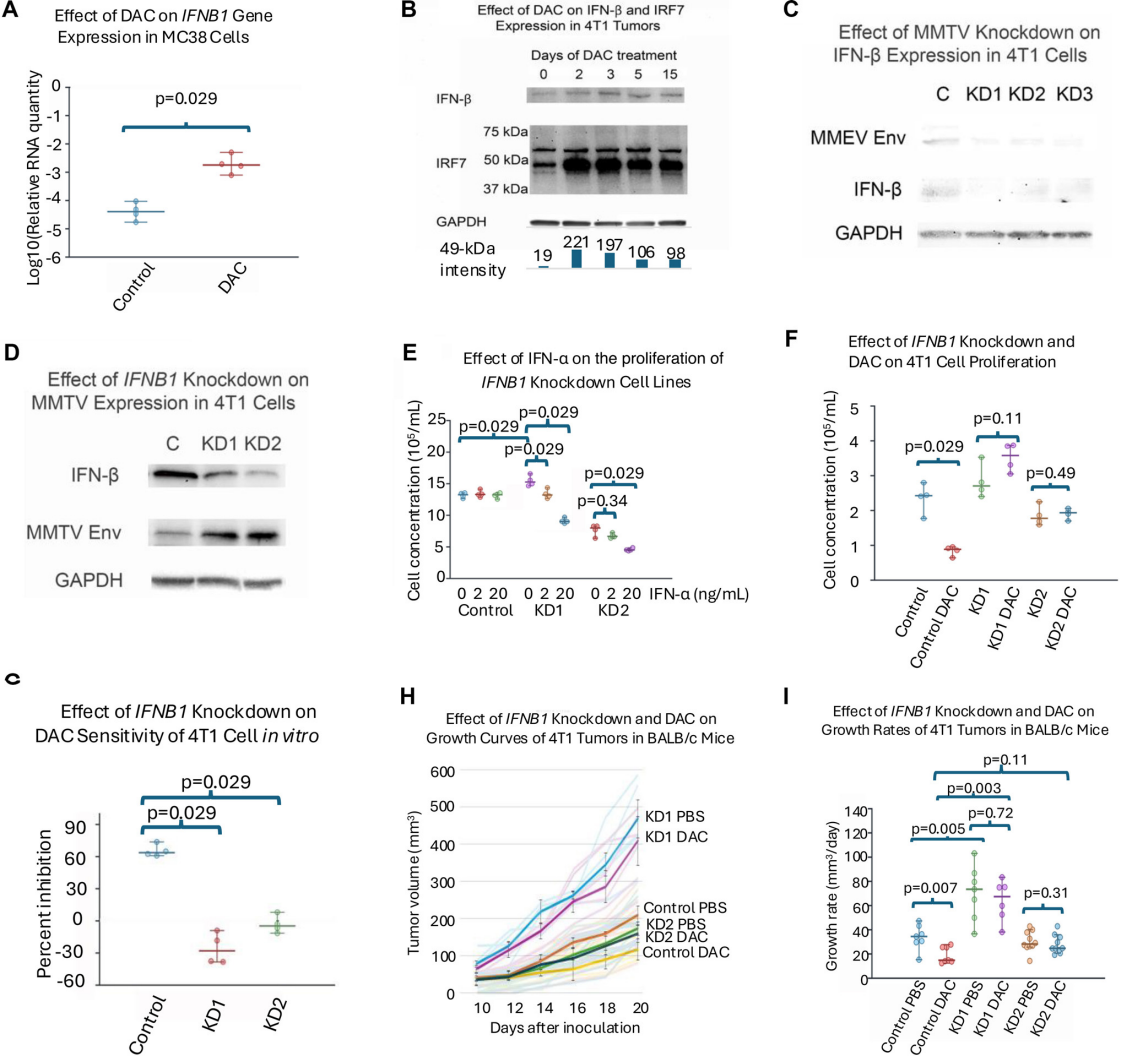
**IFN-β mediates the antitumor effect of DAC and reciprocally regulates MMTV expression.** (A) DAC induces transcriptional upregulation of *Ifnb1* in MC38 cells. Expression was quantified by qRT-PCR; (B) DAC treatment increases IFN-β and IRF7 protein levels in 4T1 tumors. BALB/c mice were treated daily for 5 days and every other day thereafter. Protein abundance was analyzed by immunoblotting. Normalized band intensities are shown below the blots; (C) MMTV knockdown reduces IFN-β protein expression in 4T1 cells. Western blot analysis of KD1–KD3 knockdown lines compared with control; (D) Knockdown of *Ifnb1* increases MMTV Env expression in 4T1 cells. Western blot showing reciprocal regulation of IFN-β and MMTV Env; (E) Effect of IFN-α on the proliferation of *Ifnb1* knockdown cell lines; (F) Effect of *Ifnb1* knockdown and DAC treatment on 4T1 cell proliferation. Viable cell counts were determined by NucleoCounter; (G) *Ifnb1* knockdown reduces sensitivity of 4T1 cells to DAC *in vitro*. Percent inhibition was calculated relative to untreated controls; (H) Tumor growth kinetics of control and *Ifnb1* knockdown 4T1 tumors in BALB/c mice treated with PBS or DAC. Tumor volumes were measured longitudinally after inoculation; (I) Growth rate analysis of 4T1 tumors following *Ifnb1* knockdown and DAC treatment. Pairwise comparisons included untreated and DAC-treated groups, as well as untreated tumors from control vs KD1. Values represent medians with 95% confidence intervals. *n* ═ 4 for *in vitro* proliferation assays (A, E–G); *n* ═ 6–7 for control and KD1, and *n* ═ 10 for KD2 *in vivo* studies (H and I). Abbreviations: DAC: Decitabine; IFN: Interferon; IFN-β: Interferon beta; IFN-α: Interferon alpha; IRF7: Interferon regulatory factor 7; GAPDH: Glyceraldehyde 3-phosphate dehydrogenase; MMTV: Mouse mammary tumor virus; Env: Envelope protein; KD: Knockdown; PBS: Phosphate-buffered saline; qRT-PCR: Quantitative reverse transcription polymerase chain reaction.

DAC treatment significantly enhanced the expression of IFN-β in mouse tumor cell lines at both the RNA and protein levels (Figure 3A and 3B). This increase was accompanied by a rapid rise in IRF7 expression immediately following DAC treatment, which subsequently declined over the treatment period (Figure 3B). A Mann–Kendall test confirms the downward trend in IRF7 protein levels from day 2 to day 15 with 96% confidence. Additionally, the knockdown of MMTV in 4T1 cells resulted in a corresponding decrease in IFN-β expression (Figure 3C), while knockdown of IFN-β led to an accumulation of MMTV Env (Figure 3D). These findings underscore a critical reciprocal relationship between MMTV and IFN-β expression.

### IFN-β knockdown conferred resistance to DAC

Lentiviral plasmids expressing shRNA to target various splice variants of the *Ifnb1* gene were employed to knock down *Ifnb1* in 4T1 cells (see Table 1 for the knockdown efficiencies of the KD1 and KD2 cell lines utilized in subsequent experiments). The knockdown of IFN-β increased the resistance of 4T1 cells to DAC *in vitro* and *in vivo* (Figure 3F–3I). Notably, untreated KD1 tumors exhibited accelerated growth compared to untreated control tumors, suggesting a tumor-suppressive role for IFN-β. To verify that the enhanced growth of KD1 tumors was attributable to the knockdown of an IFN-I, we treated both control and knockdown cell lines *in vitro* with recombinant interferon-α2 (IFN-α2). The growth of both knockdown cell lines significantly decreased in the presence of low concentrations of IFN-α2, while the control cell line remained unaffected (Figure 3E). These findings indicate that *Ifnb1* knockdown heightened the sensitivity of 4T1 cells to exogenous IFN-α2. The reduced growth of KD2 is likely a consequence of off-target effects from the lentiviral insertions.

## Discussion

This study represents the first examination of the viral mimicry hypothesis within murine tumor models. Our findings indicate that DAC effectively suppresses tumor growth and metastasis in mice, with this effect being at least partially mediated by MMTV and IFN-β.

### Role of viral RNA and proteins

The expression of viral RNA, particularly dsRNA, can elicit an antiviral response through interferon signaling [21]. Consistent with prior research on human ERVs [11], treatment with DAC resulted in significantly greater increases in MMTV RNA transcription compared to MMTV protein production. This discrepancy between RNA transcripts and protein synthesis is likely attributed to interferon-stimulated genes, such as the Zinc Finger Antiviral Protein (ZAP) and RNA-activated protein kinase (PKR), which inhibit retroviral translation [22]. Notably, we observed a correlation between the quantity of MMTV Env protein and the antineoplastic effects of DAC. This finding suggests that MMTV protein may play a role in tumor suppression as a neoantigen [9].

### Role of IFN-β in controlling viruses and tumors

While IFN-α and IFN-γ are predominantly produced by specialized immune cells, IFN-β is synthesized by nearly all nucleated cells during viral infections. This study demonstrates that knockdown of IFN-β results in the accumulation of the MMTV Env protein, thereby underscoring the antiviral function of IFN-β. Furthermore, IFN-β seems to inhibit tumor growth even without DAC stimulation, as one of the IFN-β knockdown cell lines (KD1) formed tumors more rapidly than the control cells.

### The relationship between IFN-β and IRF7

Upregulation of IRF7 expression has been recently documented in cell cultures treated with DAC as well as in tumor tissues from DAC-treated patients [23, 24]. Our findings corroborate these observations. Mice are known to exhibit high levels of IRF7, which enhances the production of various proinflammatory cytokines [25]. IRF7 serves as a critical transcription factor that mediates the downstream effects of pattern-recognition receptor signaling, facilitating the production of IFN-I [24, 26]. Notably, IFN-I also activates IRF7 expression via the JAK-STAT pathway, thereby establishing a positive feedback loop [27–29]. Consistent with this reciprocal stimulation, we observed a simultaneous increase in IFN-β and IRF7 in DAC-treated cells and tumors. Given that IRF7 is expressed by multiple cell types within the tumor microenvironment, including plasmacytoid dendritic cells, monocytes, B cells, and fibroblasts [29–31], it is likely that the tumor mesenchyme plays a significant role in this positive feedback loop.

### Downregulation of interferon response in prolonged treatment

**Figure 4.**
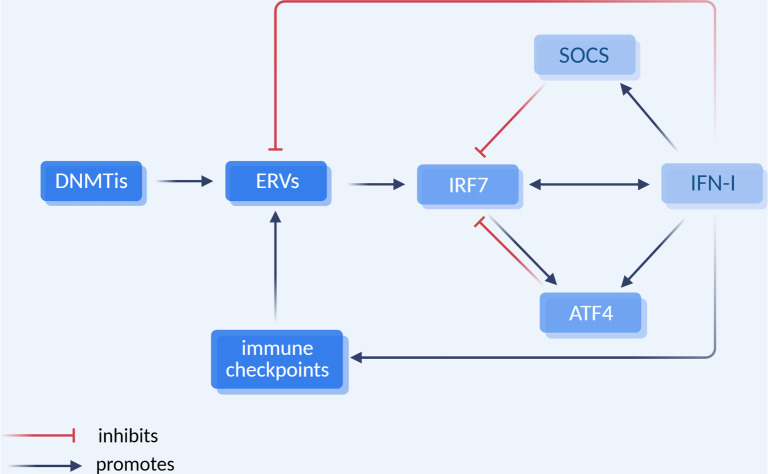
**The interplay between DNMTis, ERVs, IFN-I, and immune checkpoints.** DNMTis activate ERVs which, in turn, activate IRF7 and IFN-I. The latter two mutually stimulate each other. IFN-I inhibits both ERV expression and tumor growth. SOCS and ATF4 serve as mediators of negative feedback for the interferon system. Chronic expression of IFN-I activates immune checkpoints, leading to uncontrolled ERV expression and tumor growth. Abbreviations: DNMTis: DNA methyltransferase inhibitors; ERVs: Endogenous retroviruses; IRF7: Interferon regulatory factor 7; IFN-I: Type I interferons; SOCS: Suppressors of cytokine signaling; ATF4: Activating transcription factor 4. [Created in BioRender. Liu (2025) https://BioRender.com/tx85roj].

The observed downregulation of IRF7 during prolonged DAC therapy may elucidate the diminished effects of DAC after several weeks of treatment (Figures 1A, 2C, and 3H). Several potential mechanisms may suppress responses to IFN-I, including the downregulation of the interferon receptor (IFNAR), the induction of negative regulators, and the expression of miRNAs [32]. Additionally, certain molecules can significantly downregulate IRF7. For instance, both IFN-I and IRF7 stimulate the expression of activating transcription factor 4 (ATF4), which subsequently binds to and inactivates IRF7 [33] (Figure 4). Furthermore, IFN-I induces the expression of suppressor of cytokine signaling (SOCS) proteins that promote the degradation of IRF7 [34]. While the modulation of interferon responses is essential for preventing interferon-mediated autoimmunity, it may also compromise the efficacy of DAC therapy and other cancer treatments that depend on functional interferon responses. Conversely, in the *Ifnb1* knockdown cell lines, the interferon response system, including IFNAR, may be upregulated as a compensatory mechanism, which may explain their increased sensitivity to exogenous IFN-α (Figure 3E).

### DAC, interferons, and T cells

IFN-I inhibits tumor growth through both direct antiproliferative and immunomodulatory effects. Mechanistically, interferons downregulate oncogene expression, induce tumor suppressor genes, promote tumor cell apoptosis, enhance the expression of MHC molecules, inhibit angiogenesis, activate T lymphocytes and natural killer (NK) cells, stimulate dendritic cells, and facilitate other tumor-suppressive functions [35]. Notably, the efficacy of DAC in nude mice, which lack T lymphocytes, compared to immunocompetent mice indicates that T lymphocytes do not play a primary role in its mechanisms of action. However, the observed synergy between DAC treatment and adoptive T cell immunotherapy in mice suggests a T cell-activating effect of DAC in immunocompetent hosts [7].

### Other transposable elements and direct upregulation of IFN

Beyond ERVs, DAC activates other transposable elements such as LINE-1 in human cells [36]. Additionally, it directly demethylates the promoter region of the *IFNB1* gene [37]. These mechanisms are likely present in murine cancer cells; however, the marked decrease in IFN-β observed with MMTV knockdown (Figure 3C) underscores the critical role of MMTV in regulating IFN production within the 4T1 cell line.

### The paradoxical role of ERVs in cancer

MMTV has homologues in the human genome, specifically the human ERV type K (HERV-K) family; however, these are more degraded and incapable of horizontal transmission [38]. Driven by sex hormones, HERV-Ks are upregulated in breast cancer cells [39], with expression levels positively correlated with disease progression and unfavorable prognosis [40]. Consequently, the antineoplastic effect of DAC, which stimulates ERV expression, appears paradoxical. This paradox may be explained by negative feedback mechanisms and immune checkpoint regulation (Figure 4). DNMTi-activated viral mimicry induces the production of antineoplastic interferon type I (IFN-I). However, the duration of virus-induced interferon production is limited due to negative feedback [41]. While DAC may prolong interferon production through direct demethylation of the *IRF7* promoter [24], chronic interferon production in cancer patients can lead to the upregulation of immune checkpoint molecules, such as programmed death-ligand 1 (PD-L1) [42]. Once the interferon system is exhausted or the patient develops immunotolerance, the tumor-promoting effects of ERVs may prevail. It is plausible that high levels of ERV expression and tumor progression are both outcomes of immunotolerance. Therefore, combining interferon-based therapies with immune checkpoint blockade has demonstrated promising results [43], a strategy that may also be effective when applied to DNMTis [44].

### Limitations of the study

Some quantitative analyses may lack sufficient power to detect statistical differences due to small sample sizes (Table 2). Although DAC likely increases MMTV *pol* gene expression in 4T1 cells, similar to its effects in MC38 cells, the results for 4T1 cells did not achieve statistical significance (Figure 1E and 1F). Furthermore, 4T1 cells with MMTV knockdown (KD1) may retain some sensitivity to DAC *in vitro*, as observed *in vivo*. The discrepancy between Figure 2A and Figure 2D is likely attributable to the smaller *in vitro* sample size and the shorter duration of *in vitro* treatment (3 days *in vitro* compared to 4 weeks *in vivo*). Conversely, statistical differences observed in low-power experiments suggest large effect sizes.

**Table 2:** Post hoc calculation of achieved power for *in vitro* comparisons of knockdown cell lines

**Figure**	**Power**
2A for DAC treatment of control samples	0.94
2A for DAC treatment of KD1 samples	0.27
2B for percent inhibition difference	0.71
3E for control and KD1 cell growth	1
3E for IFN-α on KD1 at 2 ng/mL	0.91
3E for IFN-α on KD1 at 20 ng/mL	1
3E for IFN-α on KD2 at 2 ng/mL	0.34
3E for IFN-α on KD12 at 20 ng/mL	0.99
3F for DAC treatment of control samples	0.9
3G for percent inhibition differences	1

Abbreviations: DAC: Decitabine (5-aza-2′-deoxycytidine); KD: Knockdown (KD1, KD2 denote distinct knockdown lines); IFN-α: Interferon alpha.

## Conclusion

In agreement with the viral mimicry hypothesis, our studies demonstrate the involvement of the MMTV and IFN-β in mediating the antineoplastic action of DAC. There is also an upregulation of IRF7 by DAC in the murine model. A subsequent decline in IRF7 expression suggests negative feedback in the action of IFN-β and provides an explanation for the eventual failure of DAC in cancer treatment. Because interferons are known to activate immune checkpoint control, this study strengthens the theoretical basis for combining DNMTis with immune checkpoint blockers.
